# Multitask ST-LSTM model based on UAV hyperspectral remote sensing for wheat yield prediction

**DOI:** 10.3389/fpls.2026.1911934

**Published:** 2026-08-24

**Authors:** Yumeng Li, Chunying Wang, Junke Zhu, Qinglong Wang, Zengyue Li, Ping Liu

**Affiliations:** 1 School of Mechanical and Electronic Engineering, Shandong Agricultural University, Taian, China; 2 State Key Laboratory of Wheat Improvement, Shandong Agricultural University, Taian, Shandong, China; 3 School of Agricultural and Food Engineering, Shandong University of Technology, Zibo, China

**Keywords:** data augmentation, multitask learning, UAV hyperspectral, winter wheat, yield prediction

## Abstract

Timely and accurate acquisition of wheat yield is essential for ensuring food security and improving breeding efficiency. However, temporal variation relationships across growth stages have not been fully exploited in existing wheat yield prediction methods. Moreover, prediction accuracy and stability are easily affected by environmental factors and distribution shifts across different yield levels. To address these limitations, this study proposed a multitask stage temporal long short-term memory (ST-LSTM) model. A spectral- and temporal-level data augmentation method was first applied in the proposed framework to preserve the characteristics of wheat growth stages while expanding the limited sample size. Furthermore, wheat growth trend information was incorporated into the feature selection process, with both local hyperspectral features and temporal dependencies across growth stages taken into account. Subsequently, a multitask architecture combining variety classification and yield prediction was constructed, and a yield constraint was introduced to enhance the consistency between predicted yield and yield-level structure. In this framework, the yield-level probabilities learned from the variety classification task, stage-temporal features, and prior knowledge of yield distribution were jointly used to optimize the yield prediction task. Experiments on two-year datasets and an independent third-year dataset showed that the proposed model outperformed single-task models and conventional temporal models in both variety classification and yield prediction, while maintaining good stability in cross-year prediction.

## 1 Introduction

Wheat is one of the world’s major staple crops and plays a fundamental role in sustaining global food security and agricultural development (Curtis and Halford, 2014). With continued population growth, the stable improvement of wheat productivity remains a central requirement for future food supply (Lobell et al., 2011). At the same time, increasing climate variability and the more frequent occurrence of extreme weather events have intensified the uncertainty of wheat production, thereby posing substantial challenges to yield stability and food-system resilience (Wheeler and von Braun, 2013). In addition, wheat varieties differ markedly in genetic background, physiological traits, and environmental sensitivity, all of which jointly influence yield formation and its stability across environments (Pauli et al., 2016; Tattaris et al., 2016). Consequently, the efficient identification and breeding of wheat varieties with both high yield potential and strong stability has become increasingly important for ensuring food security and strengthening the seed industry (Shiferaw et al., 2013).

Accurate yield acquisition is a prerequisite for achieving this goal. However, in breeding trials and plot-scale experiments, direct yield measurement still largely depends on destructive harvesting or labor-intensive field investigation, which is often time-consuming, costly, and difficult to scale efficiently (Yang et al., 2017; Xie and Yang, 2020). These limitations are particularly pronounced under large experimental populations, where conventional phenotyping approaches struggle to meet the needs of high-throughput screening (Danilevicz et al., 2021; Hein et al., 2021). Recent advances in UAV-based plant phenotyping have improved plot-scale trait acquisition efficiency and have provided new opportunities for breeding-oriented field research (Guo et al., 2021; Volpato et al., 2021). Nevertheless, efficient data acquisition strategies and reliable yield prediction methods are still urgently needed for large-scale plot-based wheat research and breeding applications (Adak et al., 2023; Fu et al., 2022).

Remote sensing technology provides a promising solution because of its non-destructive nature, high efficiency, high throughput, and capacity for multi-scale observation (Tanaka et al., 2024). Among the sensors currently used in UAV-based agricultural monitoring, hyperspectral sensors are particularly attractive because they provide contiguous narrow bands and rich spectral detail over a wide spectral range (Ram et al., 2024). Such spectral continuity enables hyperspectral imaging to capture subtle variations associated with crop biochemical composition and physiological status (Gitelson et al., 2003; Gitelson and Merzlyak, 1994). Early studies on high-spectral-resolution canopy observation further demonstrated the potential of fine spectral information for vegetation characterization (Guyot and Baret, 1988). In wheat research, UAV mounted hyperspectral sensing has shown strong potential for yield estimation and related trait analysis at the plot scale (Tao et al., 2020; Li Z. et al., 2022), and recent breeder-oriented studies have further confirmed its value for yield-related phenotyping (Kaur et al., 2024).

Traditional hyperspectral yield estimation methods have mainly relied on empirical vegetation indices, such as NDVI, EVI, and red-edge-based indices, to describe canopy vigor, chlorophyll status, or photosynthetic activity (Rouse et al., 1974; Huete et al., 2002). These indices have been useful in many agronomic applications, and red-edge feature extraction methods have also contributed to hyperspectral trait analysis (Cho and Skidmore, 2006). However, such approaches usually exploit only a limited number of spectral bands and therefore fail to fully utilize the continuous and information-rich nature of hyperspectral data. More importantly, in multi-stage crop monitoring, the spectral regions most sensitive to yield may vary across growth stages. This makes fixed-form spectral indices less adaptable to temporally dynamic crop responses and limits their ability to characterize the complex relationships between hyperspectral signals and wheat yield.

In comparison, machine learning and especially deep learning methods provide a more powerful framework for modeling high-dimensional and nonlinear spectral–yield relationships (Zhu et al., 2017; Ma et al., 2019). Deep learning has shown clear advantages in hyperspectral data analysis, particularly in feature representation and nonlinear pattern extraction (Audebert et al., 2019). Even under insufficient-sample conditions, it remains a promising direction for hyperspectral modeling, although the risk of overfitting becomes more pronounced (Wambugu et al., 2021). Recent studies have demonstrated the potential of machine learning for wheat yield estimation from remote sensing data (Cheng et al., 2022), while broader reviews have also highlighted the growing role of advanced predictive modeling in agricultural yield prediction (Jabed and Murad, 2024). However, the performance advantage of deep models often depends heavily on sufficiently large and representative training datasets. Under small-sample conditions, especially when data are high-dimensional and affected by cross-year environmental variation, deep models may learn unstable or spurious associations, which ultimately restricts their generalization ability in practical agricultural applications.

Data augmentation has therefore attracted increasing attention as an effective strategy for alleviating sample scarcity in deep learning (Hao et al., 2023). In wheat yield estimation, augmentation-based approaches have already demonstrated their potential to improve predictive performance under limited-sample conditions (Zhang et al., 2022). Nevertheless, purely data-driven deep models still often suffer from limited interpretability and weak incorporation of agronomic prior knowledge. In this context, physics-informed or physics-guided learning offers an appealing direction by incorporating physical constraints, domain knowledge, or mechanistic priors into the training process to improve reliability, interpretability, and consistency with crop growth processes (Karniadakis et al., 2021).

Therefore, this study develops a stage temporal long short-term memory (ST-LSTM) model (Hochreiter and Schmidhuber, 1997) within a multitask learning (MTL) framework to improve wheat yield prediction from UAV hyperspectral data. Unlike conventional yield prediction methods that rely solely on data-driven regression, the proposed framework integrates both temporal growth information and agronomic prior knowledge into the modeling process. Specifically, wheat varieties are first grouped according to yield levels, and the corresponding yield distribution characteristics are used as prior information in model optimization. Augmented hyperspectral data are subsequently used as model inputs. Feature selection is performed by explicitly considering the critical temporal relationships among growth stages, thereby preserving both key spectral information and stage-wise growth dynamics. In addition, yield-level classification and yield prediction are jointly optimized through multitask learning. During this process, the predicted probabilities of different yield levels are further incorporated into a yield constraint, so as to improve the consistency between the regression output and the latent yield-level structure. In this way, the proposed method not only improves the biological and agronomic plausibility of the predictions, but also enhances model stability under limited-sample conditions and strengthens robustness in cross-year prediction scenarios.

## 2 Materials and methods

### 2.1 Experimental design

**Figure 1:**
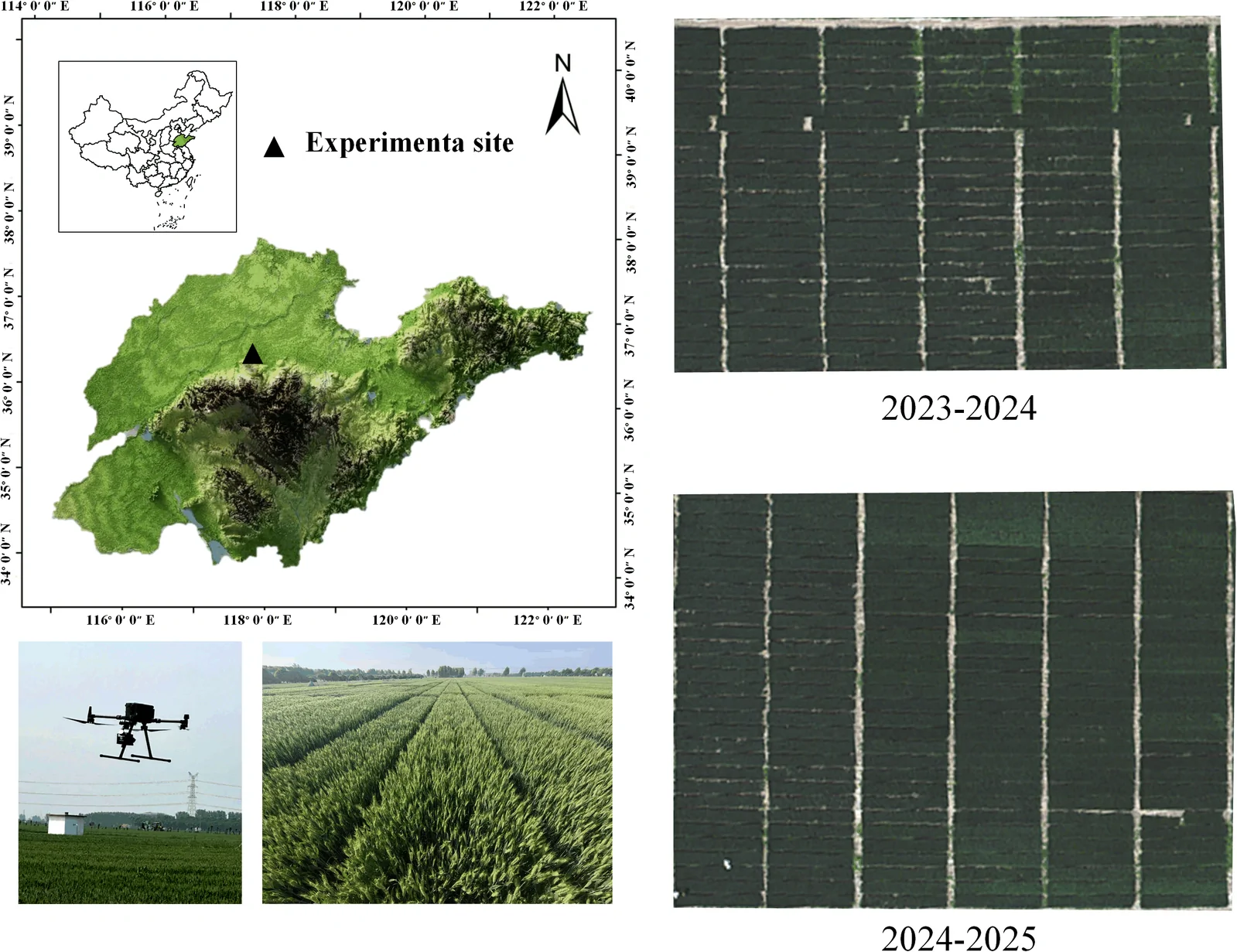
Location of the study area and spatial distribution of wheat samples in 2023–2024 and 2024–2025.

The wheat experimental field was located at HeFeng Smart Agriculture Base in Zibo City (36°56′33″ N, 118°14′42″ E). This region had a temperate monsoon climate, with an average annual precipitation of 650 mm and an annual average temperature ranging from 12.5 °C to 14.2 °C. The average annual sunshine duration was between 2209.3 hours and 2523.0 hours. The specific climatic conditions for each year from 2023 to 2026 are presented in **Table 1**. During the 2023–2026 period, all experimental wheat varieties were breeding lines developed by Zibo HeFeng Seed Company, including 197 varieties in 2023–2024, 504 varieties in 2024–2025 and 72 varieties in 2025–2026. The plots, each with an area of 10 m² (1 m × 10 m), were spaced 20 cm apart. Based on the local winter wheat planting model (with a standard nitrogen fertilizer application level of 20 kg/ha, where 60% was applied as base fertilizer and 40% was applied as top dressing at the jointing stage, with urea (N ≥ 46%) used as nitrogen fertilizer). Phosphorus and potassium fertilizers were applied entirely as base fertilizers in a single application. The experimental field followed uniform planting practices, water and fertilizer management, and pest and disease control. The location and design of the experimental field are shown in **Figure 1**.

**Table 1:** Reported climatic conditions in Zibo during 2023–2026.

Calendar year	Mean temperature (°C)	Reported maximum temperature (°C)	Reported minimum temperature (°C)	Annual precipitation (mm)
2023	15.1	41	-17	597.4
2024	15	42	-13	871.9
2025	15.3	42	-14	1054.1
2026	15	39	-17	Not available

### 2.2 Data sources

#### 2.2.1 Hyperspectral data collection

During the five important growth stages of wheat, namely the Jointing stage, Booting stage, Heading stage, Flowering stage, and Filling stage, data were collected between 11:00 and 13:00 under clear and cloud-free weather conditions to obtain stable canopy spectral reflectance data of winter wheat. Data acquisition was conducted using a DJI M300 RTK UAV equipped with a GaiaSky-mini3 hyperspectral imaging system (Dualix Spectrum). The hyperspectral imaging system covered a spectral range of 400–1000 nm, with a spectral resolution of 5.5 nm and an image resolution of 1024 × 1000 pixels. The UAV was operated at a flight altitude of 50 m and a flight speed of 5 m s^-^¹, and hyperspectral canopy data were acquired while hovering at predefined waypoints.

The acquired hyperspectral data were sequentially processed through lens correction, reflectance calibration, and atmospheric correction. The corrected images were then mosaicked and stored in TIFF format. Based on the positional and geometric characteristics of the experimental plots, an equidistant sampling method was adopted to determine the sampling regions within each plot. Specifically, the center position of each plot was first identified, and then four additional positions were selected at equal intervals on both sides as the centers of the sampling regions. To avoid edge effects, all sampling regions were located away from the plot boundaries. As a result, five sampling regions were defined for each plot, each with a length of 20 cm and a width of 60 cm. The ENVI 5.3 software was then used to extract the spectral reflectance values of each sampling region, and the mean reflectance value of the five sampling regions was calculated as the hyperspectral data of wheat for the corresponding plot.

#### 2.2.2 Yield calculation and yield-level classification

After the wheat grains reached full maturity, all experimental plots were harvested in their entirety, followed by threshing, air-drying, and weighing. Grain yield was calculated at a standard moisture content of 13% and then converted to yield per ha (kg/ha).

According to the criteria for “high-yielding and stable-yielding varieties” specified in the official wheat variety approval documents, wheat varieties were classified based on their yield performance relative to the control cultivar Jimai 22. Specifically, varieties with average yield in regional trials at least 3% higher than that of the control were classified as high-yielding varieties. Varieties with yields lower than the minimum yield of the control cultivar (Jimai 22) were classified as low-yielding varieties, while the remaining varieties were classified as conventional varieties.

### 2.3 ST-LSTM-based multitask learning model

#### 2.3.1 Data augmentation

The data augmentation methods used in this study were divided into two categories: spectral-level augmentation and temporal-level augmentation. The spectral-level augmentation methods included Gaussian noise addition, spectral scaling and spectral shifting.

Gaussian noise addition was performed by superimposing slight random Gaussian noise onto the original hyperspectral reflectance data for each sample with a probability of p=0.7. This was intended to simulate the uncertainty introduced during remote sensing observations by sensor noise, environmental interference, and measurement errors. Without altering the overall spectral shape or physical meaning, this method improved the model’s robustness to sensor noise and environmental disturbances and prevented the model from overfitting to overly clean spectral patterns.

Spectral scaling was applied to the spectrum of each growth stage with a probability of p=0.5 by multiplying the entire spectrum of that stage by a scaling factor. This operation simulated changes in reflectance magnitude caused by differences in illumination conditions, atmospheric conditions, or observation angles. While preserving the spectral variation trend, it modified the amplitude and improved the model’s adaptability to variations in illumination intensity.

Spectral shifting was mainly designed to simulate spectral feature variations caused by differences in growth stage duration. This method reduced the model’s dependence on the exact band positions of specific spectral peaks. For each growth stage, with a probability of p=0.2, the spectrum was randomly shifted from three bands to the left to three bands to the right, and missing bands were filled using nearest-neighbor interpolation.

To ensure that the augmented spectral data remained within a reasonable physical range, the application probabilities and perturbation magnitudes of all augmentation operations were strictly constrained.

The temporal-level data augmentation method fully exploited the structural characteristics of multi-stage hyperspectral observations. Considering that crop growth processes and spectral responses may vary under different plots and environmental conditions, this study introduced a stage reorganization augmentation strategy among samples of the same category. Without violating the consistency of yield labels, this strategy enhanced the diversity of temporal samples and further improved the model’s ability to capture cross-stage variations and intra-sample temporal differences. To ensure the reliability of yield labels under hard-constraint conditions, stage reorganization augmentation was performed only within the top 20% and bottom 20% subsets of samples ranked by yield.

Specifically, two augmentation approaches were adopted. The first approach reorganized spectra from different growth stages between adjacent samples of the same category after ranking, thereby constructing new stage temporal samples. The second approach generated new samples by performing linear interpolation between two complete stage temporal sequence samples while keeping the sample object unchanged. This was intended to simulate differences in within-stage variation of wheat growth under different plot or year conditions.

All data augmentation operations were applied only during the model training stage, whereas the test set data remained in their original form to ensure the objectivity and fairness of model performance evaluation.

#### 2.3.2 Feature selection method based on stage temporal information

To preserve both the intrinsic continuity of spectral reflectance along the wavelength dimension and the temporal order of wheat growth and development, the hyperspectral data of each plot, consisting of 448 bands acquired at five growth stages, were organized into a 5 × 448 two-dimensional matrix. Directly using the original high-dimensional spectra as model input may introduce redundant information, noise interference, and overfitting, especially under small-sample conditions. Therefore, it was necessary to identify the key bands most relevant to the yield prediction task while reducing the dimensionality of the spectral input.

Unlike conventional methods that independently select bands for each growth stage and subsequently merge the selected subsets, this study emphasized the stage-wise evolutionary pattern of band importance throughout the entire wheat growth process. Specifically, band selection was designed to follow the temporal continuity of crop development rather than treating each growth stage as an isolated feature-selection problem. On this basis, a feature selection strategy based on a stage-temporal spectral encoder was developed, so that the generated band-selection mask could better conform to the temporal progression of wheat growth and thereby reduce the instability caused by independent stage-wise selection.

To alleviate spectral noise and the local correlations among adjacent bands, a 1D-CNN (LeCun et al., 1998) was applied to the 448 original bands of each growth stage for local neighborhood feature fusion. This process did not alter the band dimensionality; instead, the CNN output remained 448 features corresponding to the original band positions, with each feature integrating the spectral information from its surrounding neighborhood. Therefore, the convolutional encoding did not simply produce individual single-band responses, but rather generated locally enhanced representations incorporating the information of continuous neighboring spectral regions. This enabled the model to preserve the collaborative response characteristics of locally continuous spectral segments before band selection and provided more physically meaningful inputs for subsequent band-importance evaluation.

Subsequently, the resulting stage-wise feature vectors were sequentially fed into a gated recurrent unit (GRU) according to the order of growth stages, generating hidden states that contained both the information of the current stage and the accumulated temporal information from previous stages. On this basis, a multilayer perceptron (MLP) was further used to estimate band-importance scores and generate the corresponding band-selection mask. Accordingly, the importance of each band was evaluated not only according to its spectral response at the current growth stage, but also in the context of the temporal evolution of preceding stages. This design allowed growth-trend information to be incorporated into the mask-generation process, thereby strengthening the temporal dependency in feature selection.

Compared with spatiotemporal spectral encoders, the feature selection method adopted in this study differs substantially in terms of its output form. Conventional spatiotemporal spectral encoders generally take complete full-band spectral–temporal data as input, generate compact latent representations through feature compression and nonlinear mapping, and directly use these representations for subsequent prediction tasks. In contrast, the feature selection method used in this study was applied before the ST-LSTM prediction model to select a subset of characteristic bands with high task relevance and strong interpretability from the original wavelength variables. The selected bands remained physically traceable to original wavelengths, which improved model interpretability and reduced spectral redundancy before ST-LSTM-based multitask prediction.

To improve the robustness of band selection, the above procedure was repeated 50 times under different random conditions, and the selection frequency of each band was recorded. All candidate bands were then ranked according to their selection frequencies, and the top 32 bands were retained as the final characteristic-band subset. Compared with a single-run selection strategy, this repeated-selection scheme reduced the influence of random initialization, sample partitioning, and local optima, enabling the retained bands to more reliably represent stable yield-sensitive spectral information. It should be noted that some of the final selected bands were spectrally adjacent. In the present framework, such neighboring bands should not be simply regarded as redundant, because the preceding convolutional encoding had explicitly incorporated local spectral continuity into feature extraction. Therefore, these adjacent bands were more likely to jointly represent continuous sensitive spectral regions associated with yield formation, rather than isolated single-band effects. The selected 32 characteristic bands were then used as input features for the subsequent LSTM-based multitask model for wheat yield-level classification and yield prediction.

#### 2.3.3 LSTM model based on multitask learning framework

The wheat yield prediction task in this study involved both yield-level classification and continuous yield regression. Although yield is ultimately expressed as a continuous numerical trait, wheat varieties with different yield performances still exhibit relatively clear stratification at the population level. Therefore, according to their yield performance, the samples were divided into three categories, namely high-yield, conventional, and low-yield varieties. If only the regression task is used, the model may be affected by local sample patterns, noise disturbances, and sample imbalance under small-sample conditions, and may therefore fail to fully exploit the structural differences among samples with different yield levels. In contrast, if only the classification task is adopted, the model can distinguish yield levels to some extent, but cannot provide fine-grained quantitative prediction of yield. Therefore, this study constructed a multitask learning framework so that the model could simultaneously learn both the yield-level classification task and the continuous yield regression task.

Within this framework, the stage temporal features extracted by the LSTM were used as a shared representation and were simultaneously fed into the classification branch and the regression branch. The classification branch adopted a fully connected structure to output the probabilities of the three yield-level classes, namely high-yield, conventional, and low-yield. This branch was intended to help the model learn the hierarchical differences among samples with different yield levels. The regression branch, in contrast, directly output the predicted continuous yield value, so as to preserve the model’s ability to characterize quantitative differences in yield. Since the two tasks shared the same stage temporal feature representation, the classification task could provide structural supervision for feature learning, while the regression task could maintain sensitivity to continuous numerical variation. In this way, the model was able to simultaneously exploit the category-level differences among samples and the continuous variation within categories.

Unlike a conventional multitask design in which the auxiliary task is used only to improve the shared feature representation, the classification branch in this study was also used to provide prior information for the subsequent regression constraint. Specifically, the predicted class probabilities of the three yield levels were further introduced into the yield-constrained regression process, so that the regression output was not learned independently of the yield-level structure. In this way, the multitask learning framework not only improved the stability of feature learning, but also established an intrinsic connection between yield-level classification and continuous yield prediction.

Through the above design, the classification task and the regression task were jointly optimized within a unified stage temporal representation space. The classification branch improved the model’s ability to distinguish the structural differences among high-yield, conventional, and low-yield samples, while the regression branch preserved the model’s ability to estimate the specific yield value of each sample. As a result, the model could make fuller use of both the hierarchical structure of yield levels and the quantitative variation of continuous yield, thereby improving the robustness and interpretability of wheat yield prediction under small-sample and cross-year conditions.

#### 2.3.4 Implementation details and hyperparameter settings

To improve reproducibility, the detailed network architecture and training parameters of the proposed ST-LSTM framework are summarized in **Table 2**. For each sample, the original UAV hyperspectral input consisted of five growth stages, with 448 spectral bands at each stage. After feature selection, 32 characteristic bands were retained for each growth stage. Therefore, the final input of the ST-LSTM encoder was a five-step stage-feature sequence, with 32 features at each time step.

**Table 2:** Key architecture and hyperparameters of the ST-LSTM framework.

Module	Parameter	Setting
Feature selection	Input size	5 growth stages × 448 bands
	1D-CNN local fusion	Local spectral-neighborhood fusion
	CNN kernel size	8
	GRU layers	1
	GRU hidden units	24
	MLP hidden units	32
	Activation function	ReLU
	Dropout rate	0.10
	Output	32 characteristic bands
ST-LSTM	Input size	5 growth stages × 32 selected bands
	LSTM layers	1
	LSTM hidden units	48
	Fully connected units	32
	Activation function	ReLU
	Dropout rate	0.20
	Classification branch structure	Fully connected layer + Softmax
	Output classes	Low-yield, conventional, high-yield
	Regression branch structure	Fully connected layer + linear output
	Output	Continuous yield
	Classification loss	Class-weighted cross-entropy
	Regression loss	Pseudo-Huber loss
	Constraint loss	Probability-weighted soft yield-interval loss
	Regression/classification/constraint	1.00/0.25/0.05
	Algorithm	Adam
	Initial learning rate	6 × 10^-4^
	Mini-batch size	32
	Learning-rate decay	0.55 every 80 epochs
	Gradient clipping	5.0
Cross-validation	Number of folds	10
	Maximum epochs per fold	300

The ST-LSTM prediction network consisted of a sequence input layer, an LSTM shared encoder, a dropout layer, a shared fully connected layer, and two task-specific branches. The LSTM encoder contained 48 hidden units and used the last hidden state as the shared stage-temporal representation. This shared representation was further mapped to a 32-dimensional feature vector through a fully connected layer followed by ReLU activation. The classification branch consisted of a fully connected layer and a Softmax layer to predict three yield levels, whereas the regression branch consisted of a fully connected layer with a linear output to predict continuous yield.

The model was optimized using Adam with an initial learning rate of 6 × 10^-4^ and a mini-batch size of 32. aA stepwise learning-rate decay strategy was adopted, in which the learning rate was multiplied by 0.55 every 80 epochs. Gradient clipping with a threshold of 5.0 was used to improve training stability. The total loss consisted of class-weighted cross-entropy loss, pseudo-Huber regression loss, and probability-weighted soft yield-interval constraint loss, with weights of 0.25, 1.00, and 0.05, respectively.

#### 2.3.5 Yield-constrained regression

Although wheat yield was modeled as a continuous variable in this study, its distribution was not completely unconstrained. In practice, samples belonging to different yield levels usually occupy different, although partially overlapping, regions in the yield space. Therefore, continuous yield prediction should not be regarded as a completely free regression problem, but rather as a structured prediction problem associated with yield-level distribution characteristics. If only the numerical regression error is minimized, the model may produce predictions that are numerically acceptable but inconsistent with the agronomic distribution pattern of the corresponding yield level, especially under small-sample conditions, noisy observations, and cross-year distribution shifts. To address this issue, this study introduced a yield constraint strategy into the regression branch.

Specifically, according to the yield distribution of the training data, a reasonable yield interval was defined for each of the three categories, namely high-yield, conventional, and low-yield. During model training, the deviation of the predicted yield from each class-specific interval was first evaluated separately. If the predicted yield fell within the interval of a certain class, no penalty was imposed for that class. If the predicted yield was lower than the lower bound or higher than the upper bound of that interval, a penalty was imposed according to the degree of deviation from the corresponding boundary. The predicted class probabilities output by the classification branch were then used to weight the deviations of the predicted yield from the three class-specific intervals. In this way, the contribution of each interval to the final regression constraint was adaptively adjusted according to the model’s confidence in the class assignment.

This design enabled the classification branch to provide not only category supervision, but also structured prior information for continuous yield learning. Compared with a hard interval mapping strategy, the proposed method did not directly force the regression output into a certain interval. Instead, the constraint acted in a soft and differentiable manner during training, thereby preserving the continuity of yield prediction and allowing smooth transitions for samples near the boundaries between yield levels. At the same time, because the constraint was weighted by the predicted class probabilities rather than determined only by a single hard class label, the coupling between the classification task and the regression task was further strengthened.

It should be noted that the proposed yield constraint was used only as an auxiliary regularization term during the model training stage. The final yield prediction was still directly output by the regression branch, and no additional interval clipping, label-dependent mapping, or post-processing operation was required during inference. Therefore, this design not only incorporated the prior distribution information of different yield levels into model optimization, but also avoided the inconsistency between training and inference that may be caused by explicitly using class labels to determine regression intervals.

**Figure 2:**
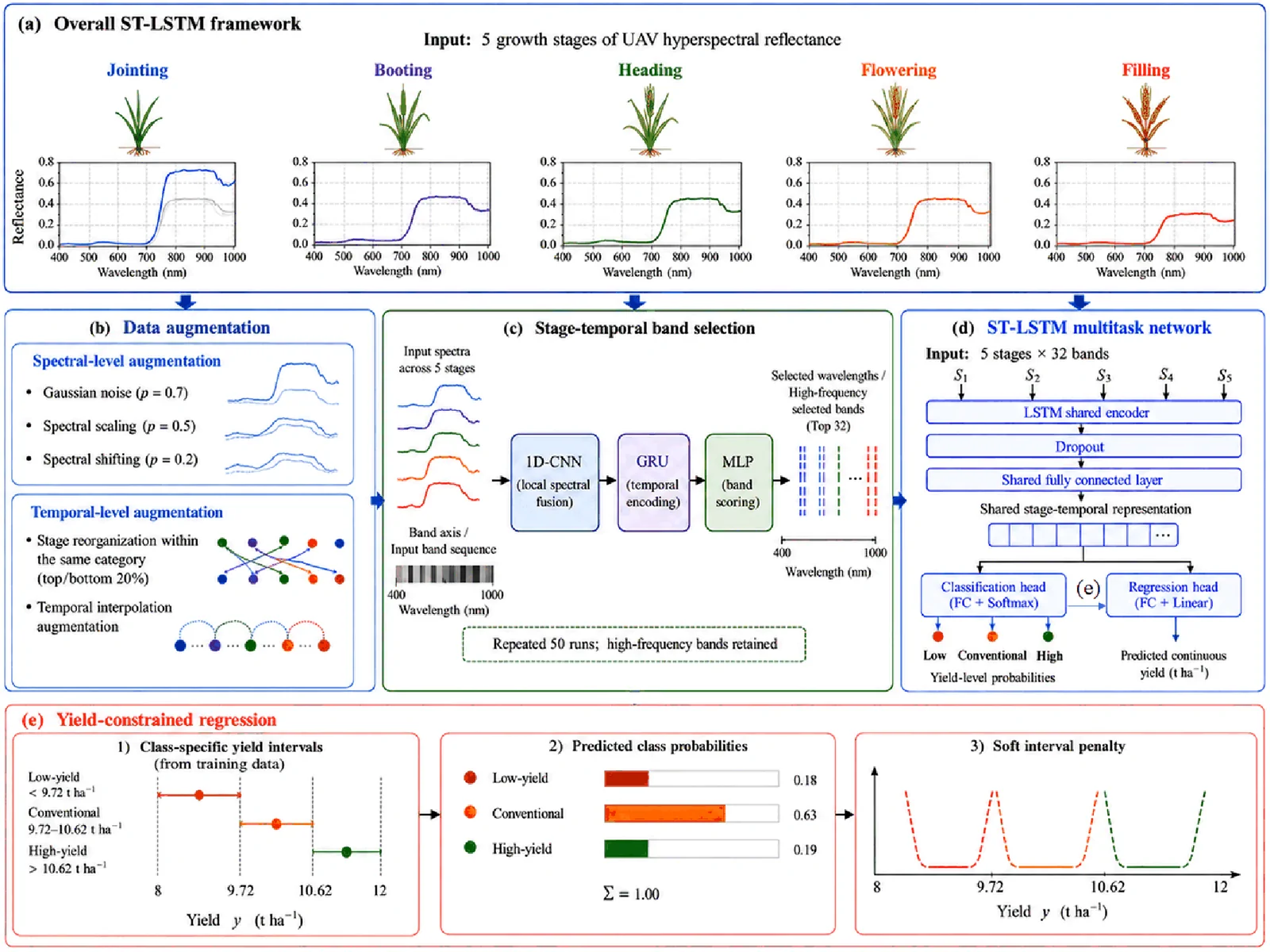
Flowchart of the multitask ST-LSTM model: **(a)** overall ST-LSTM framework; **(b)** data augmentation; **(c)** stage-temporal band selection; **(d)** ST-LSTM multitask network; and (e) yield-constrained regression.

Through the joint action of the classification loss, regression loss, and interval consistency loss, the model was able to simultaneously learn the hierarchical structure among yield levels, the numerical relationship between hyperspectral features and yield, and the distributional consistency between predicted yield and yield categories. As a result, the proposed yield-constrained regression strategy further improved the stability, plausibility, and robustness of wheat yield prediction. The overall workflow of the model is shown in **Figure 2**.

#### 2.3.6 Model performance evaluation

To comprehensively evaluate the performance and robustness of the constructed model, ten-fold stratified cross-validation was adopted in this study. The samples were first grouped according to the three yield levels, namely low-yield, conventional, and high-yield classes. Within each yield level, samples were sorted according to the measured yield and then sequentially assigned to ten folds, ensuring that each fold contained samples from different yield ranges and maintained a relatively balanced yield-level distribution.

In each round of cross-validation, nine folds were used for model training, and the remaining fold was used for validation. To avoid information leakage, the complete workflow, including data augmentation, normalization-parameter estimation, model training, and validation, was independently performed within each fold. Specifically, data augmentation and normalization-parameter estimation were conducted using only the training folds, whereas the validation fold was kept unchanged and used only for model evaluation.

For the model comparison and ablation experiments, the complete two-year dataset was used, and all models were evaluated using the same ten-fold stratified cross-validation strategy described above. This setting was used to assess the overall predictive ability of different models and the contribution of each module under the same data conditions. For the cross-year experiments, a bidirectional independent-year validation strategy was adopted. Specifically, the 2023–2024 growing-season dataset was first used for model training, while the 2024–2025 growing-season dataset was used for independent validation. The experiment was then repeated in the reverse direction. In each cross-year experiment, data augmentation, feature selection, estimation of data normalization parameters, model hyperparameter tuning, and model training were performed exclusively using data from the training year. Data from the validation year were used only to evaluate the model’s transferability under interannual variations in environmental conditions and data distributions. Furthermore, the 2025–2026 growing-season dataset was used as a completely independent test set to evaluate the generalization performance of the yield prediction model on data from an unseen year. The model was developed using only the 2023–2024 and 2024–2025 growing-season datasets. The 2025–2026 dataset was not used to generate augmented samples and did not participate in the estimation of normalization parameters or feature-selection criteria. This experimental design prevented information leakage from the independent test year.

Model performance was evaluated from both classification and regression perspectives. For the yield-level classification task, model performance was evaluated using accuracy, precision, recall, macro-F1 score, and the confusion matrix. Accuracy reflects the overall proportion of correctly classified samples. Precision indicates the reliability of samples predicted as a given class, whereas recall reflects the proportion of samples in a given true class that were correctly identified. The macro-F1 was also reported to evaluate the average classification performance across all classes without being dominated by the majority class. The confusion matrix was used to further analyze the misclassification patterns among low-yield, conventional-yield, and high-yield samples. For yield prediction, the coefficient of determination (R²), root mean square error (RMSE), and mean absolute error (MAE) were adopted. R² reflects the overall goodness of fit, RMSE emphasizes large prediction errors, and MAE represents the average magnitude of prediction deviation. The same cross-validation strategy and evaluation metrics were applied to the proposed model, baseline models, and ablation experiments to ensure fair comparison.

## 3 Results and analysis

### 3.1 Effectiveness validation of data augmentation

**Figure 3:**
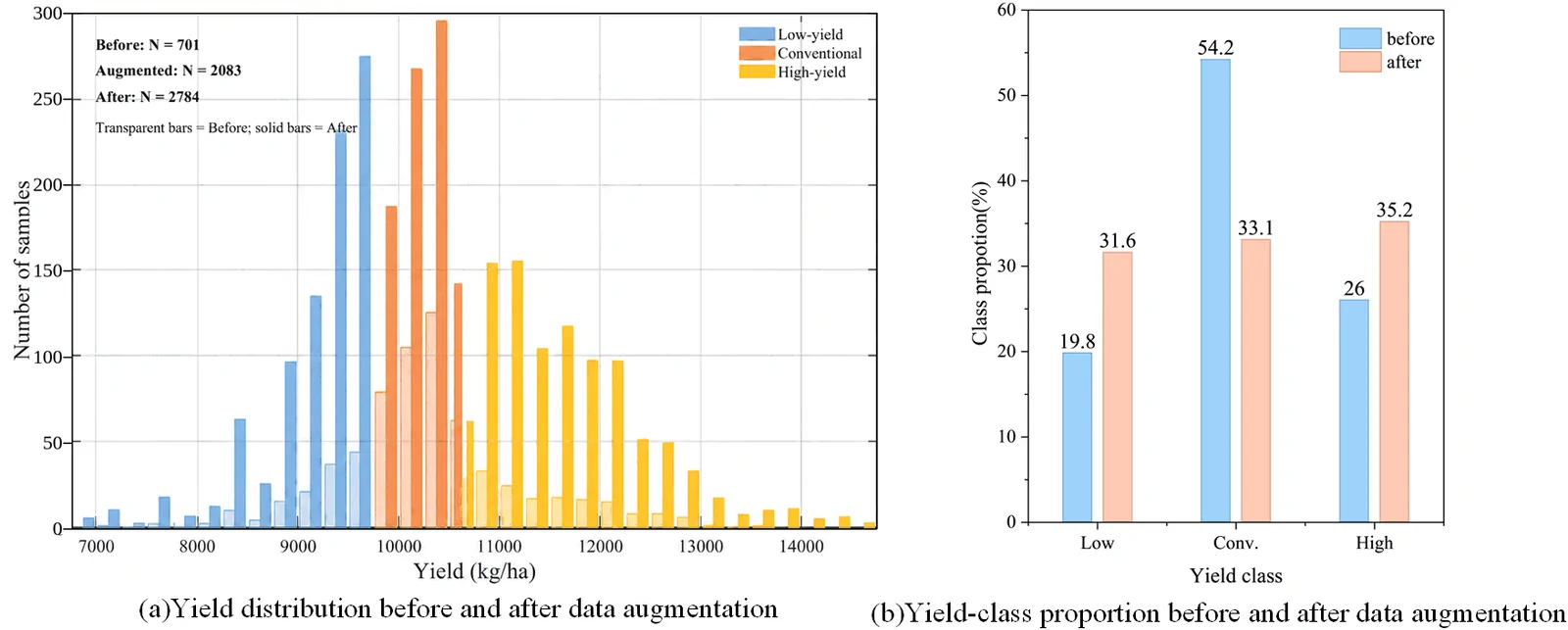
Yield distribution and yield-class composition before and after data augmentation: **(a)** yield distribution before and after data augmentation; **(b)** yield-class proportion before and after data augmentation.

To evaluate the effectiveness of the data augmentation strategy in addressing class imbalance in the classification task, the yield distribution and yield-level proportions before and after data augmentation were compared. The original dataset showed a relatively severe class imbalance, as shown in **Figure 3**, with conventional-yield varieties accounting for the largest proportion. Before data augmentation, low-yield, conventional-yield, and high-yield varieties accounted for 19.8%, 54.2%, and 26.0% of the dataset, respectively. After data augmentation, the proportions of low-yield, conventional-yield, and high-yield varieties changed to 31.6%, 33.1%, and 35.2%, respectively. Compared with the original dataset, the proportions of low-yield and high-yield samples increased, effectively improving the representation of yield levels with fewer samples and making the distribution among yield classes more balanced.

A more balanced class distribution is beneficial for training the classification branch of the multitask model. In the original dataset, the model may tend to be biased toward the majority conventional-yield class, thereby reducing its ability to identify low-yield and high-yield samples. After data augmentation, the three yield levels were more evenly represented in the training data, providing the model with training information covering different yield ranges. Therefore, the data augmentation strategy not only increased the number of training samples but also helped alleviate the class imbalance problem.

To further evaluate the physiological plausibility of augmented samples, LAI and SPAD were selected as agronomic indicators for physiological consistency assessment. Since different augmentation strategies may introduce different types of physiological risks, separate validation strategies were designed for linear interpolation augmentation and stage reorganization augmentation.

For linear interpolation augmentation, new spectral curves were generated from two source samples(P_1_,P_2_), and the main concern was whether the generated spectra still corresponded to reasonable crop phenotypic states. Therefore, LAI and SPAD inversion models were established using only original samples with measured phenotypic traits(The RF algorithm was adopted for phenotype inversion). The estimated LAI and SPAD values obtained from the augmented samples were compared with the measured phenotypic ranges of the two source samples at the same growth stage. Considering the uncertainty of phenotype inversion, a 10% tolerance range was introduced. When the estimated phenotypic value of the augmented sample fell within [0.9×min(P_1_,P_2_), 1.1×max(P_1_,P_2_)], the interpolation-augmented sample was considered to have good physiological consistency at the corresponding growth stage. This criterion was used to determine whether the spectra generated by interpolation remained within the reasonable phenotypic range defined by the two source samples.

For stage reorganization augmentation, the exchanged growth-stage spectra were directly obtained from real measurements, and therefore the exchanged stage itself already represented a realistic physiological state. Consequently, the major risk of this augmentation strategy was not whether the phenotype of the exchanged stage was physiologically reasonable, but whether the exchange operation disrupted the original growth-stage continuity. Therefore, the physiological consistency assessment of stage-exchange augmentation focused on the consistency of LAI and SPAD temporal variation trends before and after augmentation.

For each augmented sample, the phenotypic differences between adjacent growth stages of the original sample and the augmented sample were calculated. Subsequently, the changing directions of these differences were compared between the original and augmented samples. When the signs of the phenotypic differences were consistent between the two samples, the corresponding growth-stage variation trend was considered consistent, indicating that the augmented sample maintained good physiological consistency.

The results are shown in **Table 3**, and both augmentation strategies maintained high physiological consistency. For linear interpolation augmentation, the consistency rates of LAI and SPAD reached 94% and 97%, respectively. These results indicate that the interpolated spectral sequences generally remained within the reasonable phenotypic ranges defined by the two source samples. The higher consistency observed for SPAD suggests that chlorophyll-related variation patterns were well preserved during spectral interpolation, while the slightly lower LAI consistency may be attributed to the stronger sensitivity of canopy structure-related traits to spectral mixing. For stage reorganization augmentation, the trend consistency rates of LAI and SPAD were 79% and 91%, respectively. Although slightly lower than those of temporal interpolation augmentation, the results demonstrate that reorganizing growth-stage information from different samples did not substantially disrupt the temporal variation patterns of crop physiological traits. The relatively higher consistency of SPAD compared with LAI indicates that chlorophyll-related variation patterns were more stable after growth-stage recombination, whereas LAI, as a canopy structural trait, was more sensitive to changes in growth-stage combinations.

**Table 3:** Physiological consistency evaluation of augmented samples.

Augmentation strategy	Trait	Evaluation criterion	Consistency rate	
Linear interpolation augmentation	LAI	Phenotypic range consistency	94%	
Linear interpolation augmentation	SPAD	Phenotypic range consistency	97%	
Stage reorganization augmentation	LAI	Trend consistency rate	79%	
Stage reorganization augmentation	SPAD	Trend consistency rate	91%

Overall, both augmentation strategies achieved acceptable physiological consistency, indicating that the generated samples not only increased sample diversity but also maintained the major physiological variation patterns of winter wheat growth processes.

### 3.2 Comparison experiment

**Figure 4:**
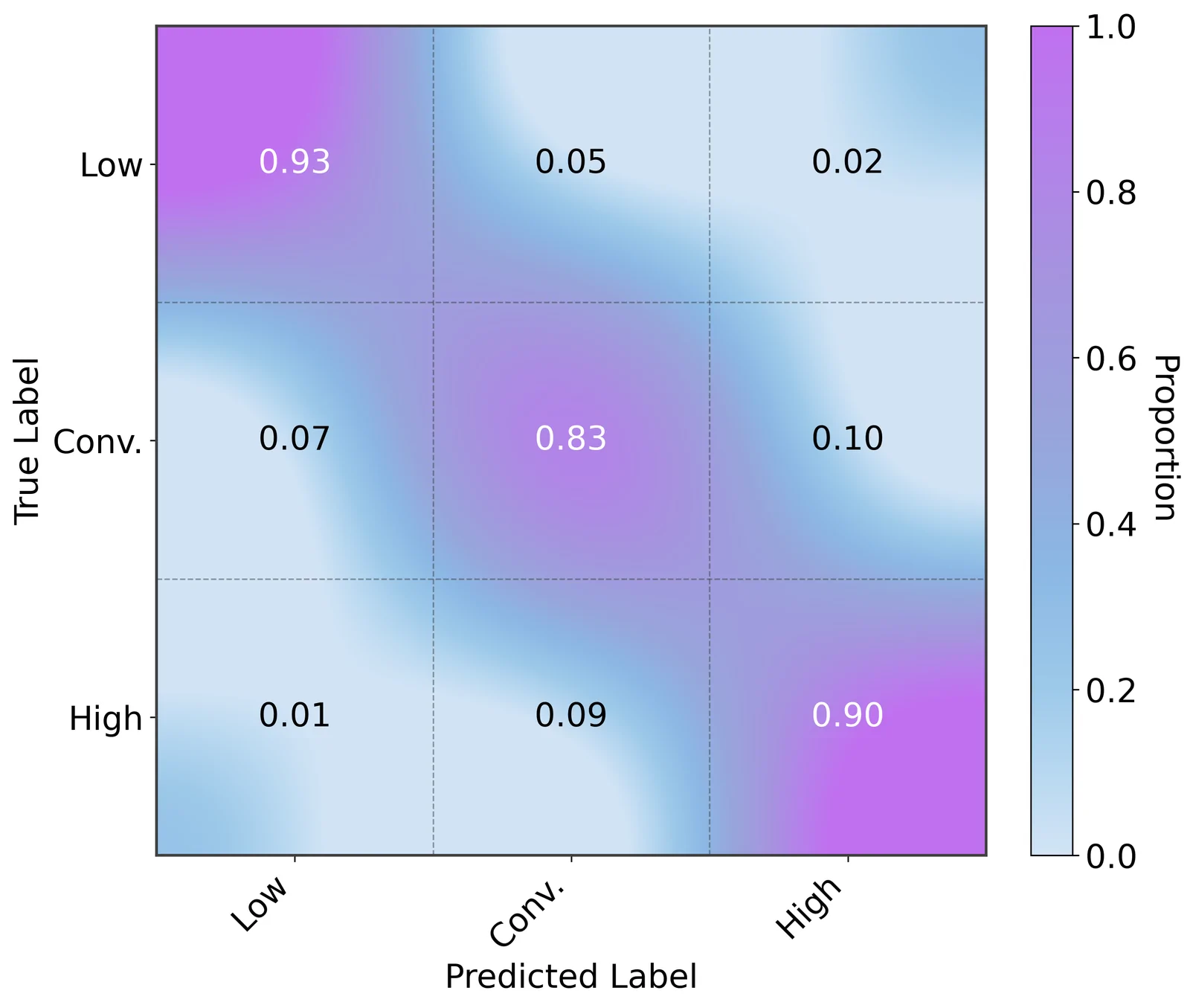
Confusion matrix of the ST-LSTM model for yield-level classification.

Under the ten-fold cross-validation framework, the proposed ST-LSTM model showed good performance in both classification and regression tasks. To avoid the influence of a single data partition on the classification results, the predictions from all validation folds in the ten-fold cross-validation were pooled, and the evaluation metrics were calculated based on the pooled results. The classification performance for different yield levels and the confusion matrix are shown in **Table 4** and **Figure 4**, respectively. The ST-LSTM model achieved an overall accuracy of 0.87 in the classification task, with macro-precision, macro-recall, and macro-F1 score values of 0.853, 0.888, and 0.867, respectively. In the regression task, the pooled R² of the ST-LSTM model reached 0.73, with RMSE and MAE values of 610.53 kg/ha and 525.21 kg/ha, respectively.

**Table 4:** Classification performance for different yield levels.

Yield class	Precision	Recall	F1-score
Low-yield	0.816	0.928	0.869
Conventional	0.932	0.833	0.880
High-yield	0.810	0.902	0.853
Macro average	0.853	0.888	0.867
Overall accuracy			0.870

**Figure 5:**
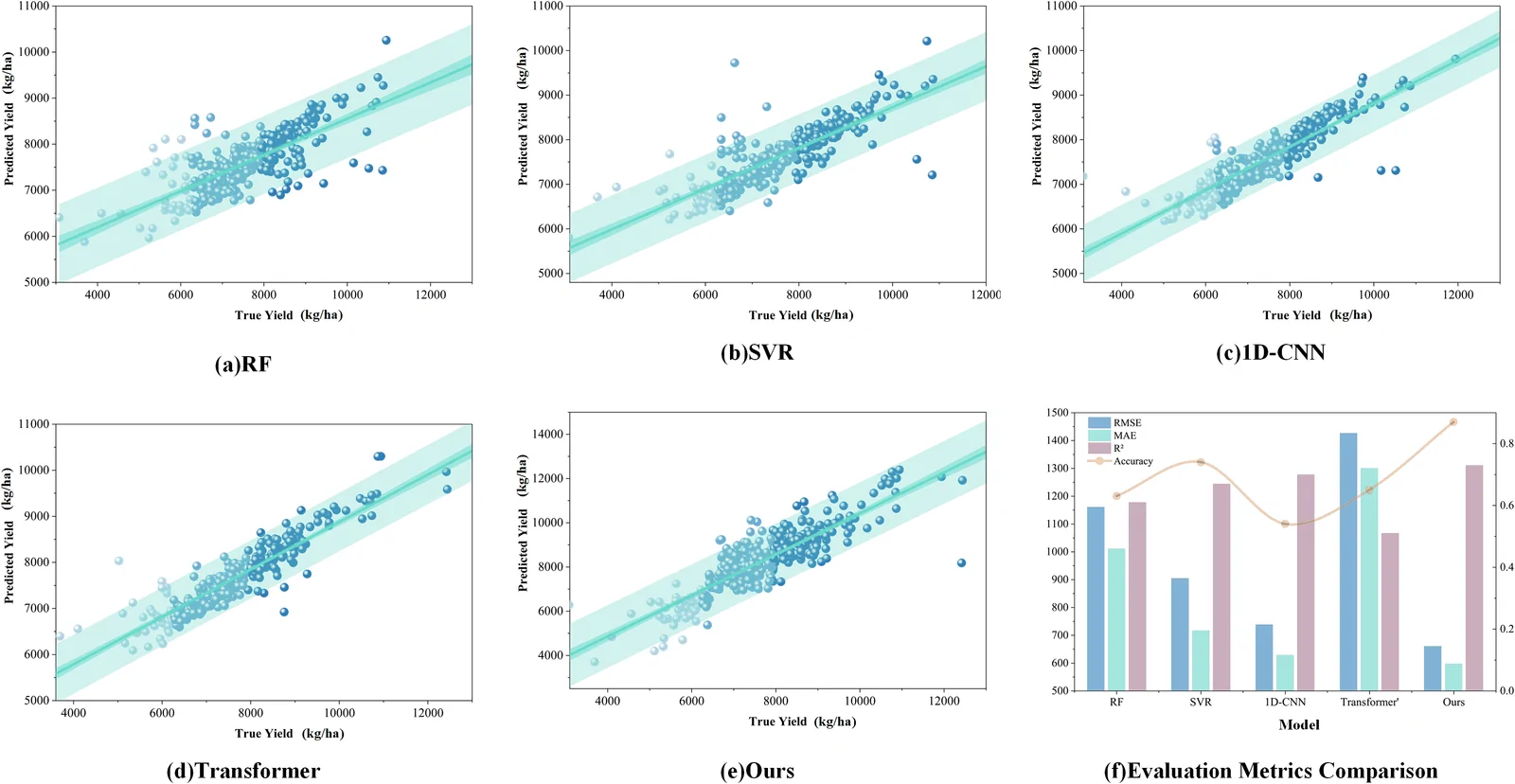
Comparison of **(a)** RF, **(b)** SVR, **(c)** 1D-CNN, **(d)** Transformer, **(e)** ours model and **(f)** Evaluation metrics comparison.

**Figure 6:**
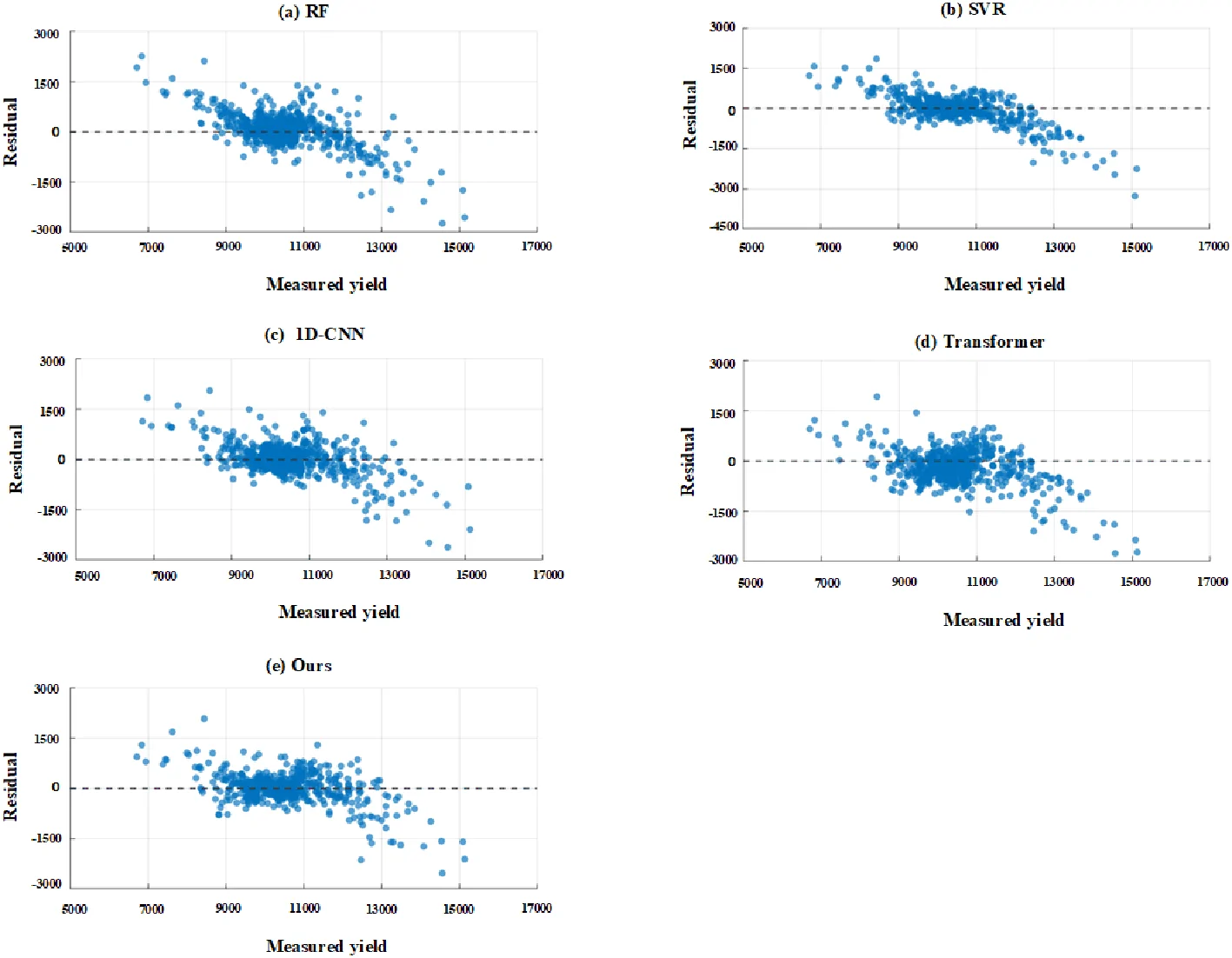
Residual comparison of **(a)** RF, **(b)** SVR, **(c)** 1D-CNN, **(d)** Transformer, and **(e)** the proposed model.

To verify the advantages of the proposed model over existing methods in terms of accuracy and stability under the same conditions, RF (Breiman, 2001), SVR (Drucker et al., 1997), 1D-CNN, and Transformer (Vaswani et al., 2017) were selected as baseline models for comparative experiments. All models adopted the same data partitioning strategy, input data, and evaluation metrics to ensure the fairness of the comparison. The comparison results and Residual are shown in **Figures 5**, **6**. In comparison, the classification accuracy and regression performance of RF and SVR were both lower than those of the proposed model. Although RF and SVR can model nonlinear relationships to some extent through tree-based structures and kernel functions, respectively, their ability to exploit the continuous band structure of hyperspectral data and the temporal variation among different growth stages is limited. Therefore, they are unable to sufficiently characterize the complex spectral-temporal response features associated with yield formation. The classification accuracy and yield prediction performance of 1D-CNN also did not reach the level of the ST-LSTM model, indicating that relying only on local spectral feature extraction is insufficient to distinguish samples from different yield levels, especially near the boundaries between adjacent yield classes where local band responses may be highly similar. Although Transformer has the ability to model long-range dependencies, its relatively lower performance may be attributed to the fact that attention-based models generally require sufficient training samples to fully capture complex dependencies. Under the small-sample and short temporal sequence conditions of this study, its structural advantages were not fully realized. In addition, the prediction errors of the ST-LSTM model were more concentrated around zero, whereas the Transformer model exhibited a wider residual distribution and more pronounced extreme errors. Compared with RF, SVR, 1D-CNN, and Transformer, the proposed model achieved better overall performance in both classification and regression tasks. For the classification task, the proposed model not only improved the overall classification accuracy but also maintained good class-wise average recognition performance under an imbalanced class distribution. For the regression task, the proposed model improved yield fitting ability while effectively reducing prediction errors.

In addition to the conventional baseline models, QRBiLSTM-MHSA (Lu et al., 2025) and CYPNet (Xiang et al., 2026) were further included as recent advanced spectral-temporal deep learning methods for comparison in the regression task. QRBiLSTM-MHSA integrates BiLSTM and multi-head self-attention to capture temporal and global dependency information from hyperspectral data, whereas CYPNet combines CNN, BiLSTM, and multi-head self-attention for multi-temporal UAV hyperspectral yield prediction. For a fair comparison, the main architectures of QRBiLSTM-MHSA and CYPNet were retained without changing their core modeling modules. Only the input layer was adapted to match the data format used in this study, so that both models could accept the original hyperspectral bands from five growth stages as input. Since these two methods were designed for continuous yield prediction and did not provide yield-level classification outputs, they were compared with the proposed ST-LSTM model only in the regression task.

The results showed that QRBiLSTM-MHSA achieved an R² of 0.666, with RMSE and MAE values of 1012.53 kg/ha and 890.06 kg/ha, respectively. CYPNet achieved an R² of 0.630, with RMSE and MAE values of 1183.55 kg/ha and 972.71 kg/ha, respectively. Both models showed lower regression performance than the proposed ST-LSTM model, whose R² reached 0.73 and whose RMSE and MAE were 610.53 kg/ha and 525.21 kg/ha, respectively. This result indicates that directly introducing complex attention-based spectral-temporal networks did not achieve the best performance under the limited-sample and high-dimensional hyperspectral conditions of this study. In contrast, the proposed ST-LSTM model further incorporated stage-aware feature representation, multitask learning, and yield-constrained regression, which jointly improved continuous yield prediction while maintaining yield-level classification ability.

### 3.3 Ablation experiment

**Figure 7:**
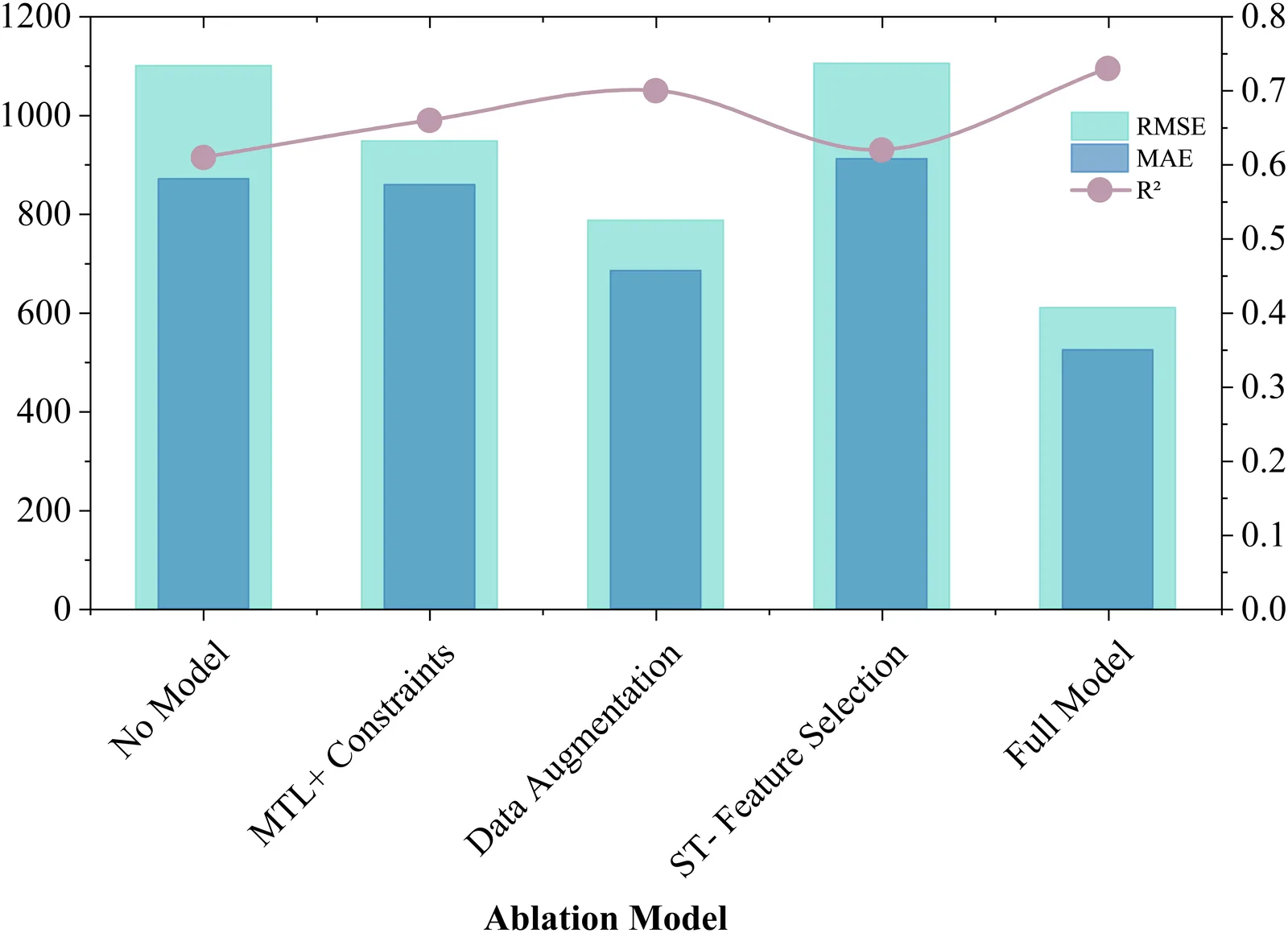
Ablation study results comparison. RMSE and MAE are displayed on the left y-axis, while R² is displayed on the right y-axis.

To verify the importance of each module, comparative experiments were conducted by applying each module individually. From a horizontal comparison, each module contributed to model performance to a different extent when used alone, and all of them effectively improved the overall model performance. The results are shown in **Figure 7**.

Among them, the model using only the data augmentation module showed the largest performance improvement, and its results were the closest to those of the full model. This demonstrates that the temporal-level data augmentation strategy plays a positive role in yield prediction. By expanding the distribution range of training samples along the temporal dimension, this strategy increased the temporal diversity of the samples, enabling the model to learn richer temporal variation patterns and improving its adaptability to samples from different environments and varieties. It also indicates that, in small-sample learning, spectral-level data augmentation is a key strategy for improving model performance. In addition to addressing the class imbalance among high-yield, low-yield, and conventional varieties in practical breeding applications, it also alleviates training bias caused by insufficient samples and enhances the generalization ability of the model.

In addition, although the models using only the MTL module and the yield-constraint module performed overall worse than the model with data augmentation, they still exhibited clear performance gains. Compared with optimization strategies conducted solely at the data level or feature level, the strategy proposed in this study—guiding the regression task through yield-level classification and further regularizing the regression output through a yield constraint—can more effectively exploit prior knowledge of yield distribution, thereby exerting a more direct effect on improving the rationality and accuracy of yield prediction. By comparing the difference between RMSE and MAE, it can be clearly observed that the model was able to simultaneously learn both the differences among yield levels and the continuous variation patterns within each level, thereby improving the consistency and stability of the predicted yield distribution.

At the feature level, the growth-stage feature module preserved the model’s dynamic understanding of the wheat growth process. While removing ineffective features, it retained important features across the growth-stage sequence, enabling the model to move beyond reliance on information from a single growth stage or local spectral segments. However, the model equipped with this module alone showed relatively weak performance, indicating that this approach could not be directly translated into stable predictive capability. Its representational advantages can only be fully realized when combined with reasonable strategies and modeling frameworks at both the data and task levels. This also explains why the full model, which integrates all modules, outperformed the models corresponding to each individual module.

To further evaluate the stability of the characteristic-band selection process, the high-frequency Top-32 bands identified from 50 repeated runs were compared with the band-selection results obtained from each of the 50 individual runs. Compared with the single-run selection strategy, the 50-run repeated-selection strategy increased R² from the minimum value of 0.45 to 0.56 and reduced RMSE from 1471.45 to 1105.58. Further comparative analysis of the selected bands showed that single-run band selection was susceptible to random initialization, sample partitioning, and convergence to local optima. In contrast, the repeated selection strategy based on selection frequency reduced uncertainty in the characteristic band identification process, retained wavelength information that exhibited more stable relationships with yield formation, and provided a more reliable input representation for the subsequent ST-LSTM model. Therefore, the repeated selection strategy not only improved the robustness of characteristic band selection but also strengthened the coordination between the growth stage characteristic module and the subsequent temporal prediction model.

The full model achieved the best results across all evaluation metrics. This further indicates that the three modules did not function independently, but rather worked together to improve model performance. Their combination enabled the model to make fuller use of stage temporal information and to generate accurate and stable yield predictions. These results further demonstrate the effectiveness of the proposed framework for wheat yield prediction.

### 3.4 Early prediction experiment

**Figure 8:**
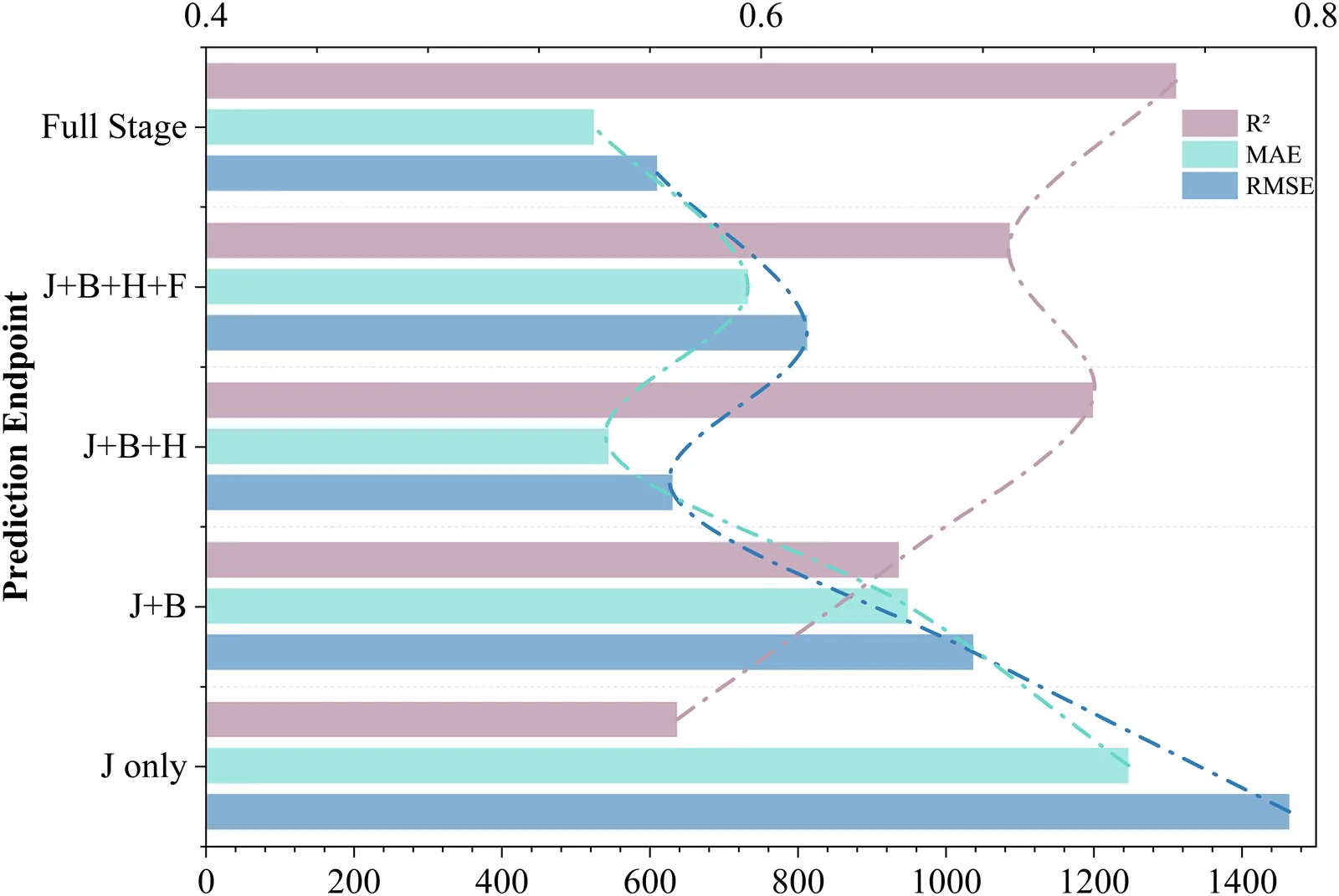
Comparison of experimental results at different growth stage endpoints. RMSE and MAE are shown on the bottom x-axis. R² is shown on the top x-axis. J, B, H, and F correspond to Jointing stage, Booting stage, Heading stage, and Flowering stage, respectively.

The results based on input from the single-stage jointing stage represented the weakest performance among the five settings. Using only single-temporal hyperspectral data from the jointing stage was insufficient to accurately characterize the final yield formation process. Although this stage is a critical transition period from vegetative growth to reproductive growth in wheat, yield-related phenotypic traits are still undergoing continuous formation and differentiation at this time, and key information directly related to final yield—such as subsequent spike development, nitrogen allocation, and grain formation—has not yet been fully expressed. However, the relatively poor performance at this stage does not imply that jointing-stage information is ineffective; rather, it indicates that single-temporal observations can provide only limited early discrimination capability and remain insufficient to fully explain final yield. The results are shown in **Figure 8**.

The results obtained under different prediction endpoints showed that model performance exhibited stage-wise fluctuations but an overall trend of significant improvement. When the input was extended from a single growth stage to a multi-stage sequence, the model was better able to capture the continuous dynamic changes during wheat growth, thereby shifting from “static representation” to “dynamic discrimination.” From a physiological perspective, stage temporal input enhanced the model’s ability to dynamically capture wheat growth rate and nutrient accumulation status, and the extracted features were more directly and stably associated with subsequent yield formation. Therefore, the use of stage temporal hyperspectral data substantially improved the model’s ability to predict wheat yield.

When the prediction endpoint was extended to the flowering stage, model performance declined to some extent, although the results still outperformed those of J only and J+B. This phenomenon may be attributed to two possible reasons. First, the flowering stage is relatively short in duration, and is more sensitive to short-term weather conditions and the timing of data acquisition, which can easily introduce additional noise. Second, the determination of the flowering stage relies on manual judgment. At the beginning and end of flowering, spectral responses may fluctuate more strongly because of differences in the degree and extent of spike exposure above the canopy, thereby increasing interference from temporal disturbances. However, after further incorporating grain-filling-stage information, the performance of the Full Stage setting improved significantly and reached the best level among the five settings. The additional noise and disturbance introduced during flowering were re-integrated within a more complete growth sequence, resulting in a smaller effect on the explanatory power for final yield. In addition, the grain-filling stage provided information most directly related to final yield, enabling the model to achieve more accurate yield prediction under the complete temporal sequence.

The stage-wise performance of yield prediction under different growth-stage endpoints further indicates that the contributions of different growth stages to yield prediction are not equivalent, and that complementary as well as interfering relationships exist among the data from different stages. On the one hand, incorporating data from multiple growth stages can significantly improve yield prediction performance. On the other hand, although some growth stages are physiologically important, their hyperspectral data may contain additional noise or environmental disturbances, and their contributions therefore need to be re-evaluated within a complete stage temporal framework. Accordingly, the practical contribution of each growth stage to yield prediction should be comprehensively assessed from both the perspective of wheat yield formation mechanisms and data quality.

Later growth stages generally provide information that is closer to final yield and therefore help improve prediction accuracy, but they also inevitably postpone the prediction timing. For studies primarily aimed at variety screening and model performance improvement, models including grain-filling stage data are more advantageous because they incorporate more complete information on yield formation. From a practical application perspective, however, the later the prediction is made, the less lead time remains for field management and yield warning, and thus its practical value is correspondingly reduced. In contrast, stage combinations ending before anthesis, although slightly less accurate than full-season models, can already provide relatively stable prediction results while substantially advancing the prediction timing, making them more suitable for application scenarios that require early decision-making. Therefore, the optimal temporal endpoint for wheat yield prediction should be determined by balancing prediction accuracy and prediction timeliness according to the specific research objective.

### 3.5 Cross-year comparative experiments

Climatic conditions, precipitation and temperature patterns, soil background, field management practices, and hyperspectral remote sensing conditions may all vary across years, resulting in stronger distribution shifts in samples from cross-year experiments. If a model relies only on local statistical characteristics from a single year, it is usually difficult to maintain stable performance in another year. Therefore, this study adopted a bidirectional validation strategy, namely “training on the first year and testing on the second year” and “training on the second year and testing on the first year,” to evaluate the cross-year generalization ability of the model.

**Figure 9:**
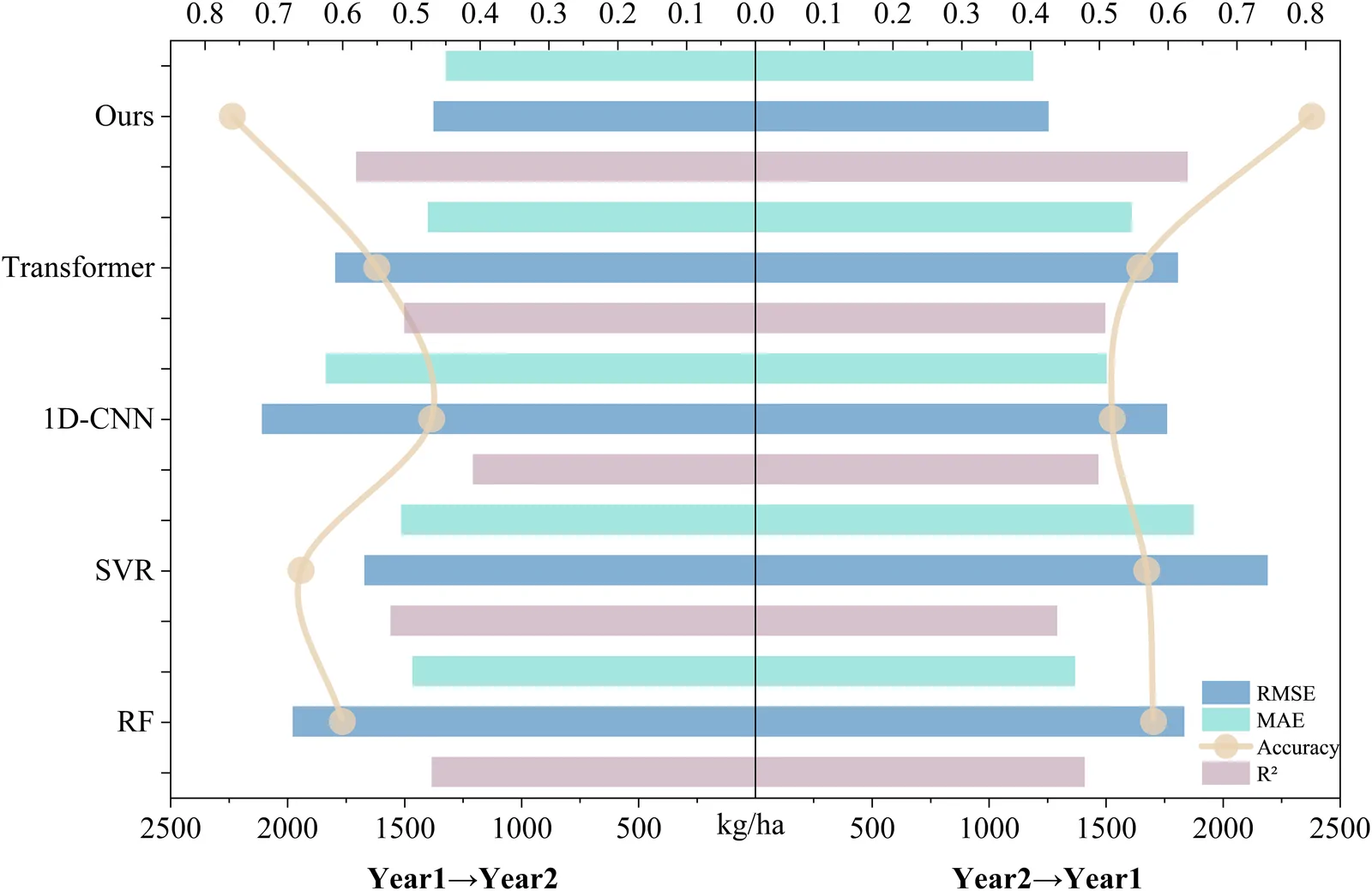
Comparison of cross-year experiments. The left and right sides of the image (separated by the center line) show model performance under two cross-validation scenarios: (left) Year1 for training and Year2 for testing; (right) Year2 for training and Year1 for testing. RMSE and MAE are displayed on the lower x-axis, while R² and accuracy are displayed on the upper x-axis.

The results of the cross-year experiments are shown in **Figure 9**. The R² values of the comparison models decreased to below 0.53, accompanied by a substantial increase in RMSE. These models depended heavily on the distributional relationship between hyperspectral data and yield within the training year, and thus exhibited limited generalization ability when distribution shifts occurred in the cross-year test sets. In contrast, the proposed model maintained relatively good R² values of 0.59 and 0.63, while the variations in RMSE and MAE remained comparatively small. Under these conditions, the proposed method consistently achieved relatively leading accuracy in both the Year1 to Year2 and Year2 to Year1 experiments, indicating that it was able not only to learn superficial correlations between hyperspectral features and yield, but also to capture more stable cross-year discriminative information to a certain extent. Among the contributing factors, the yield constraint largely prevented the model from generating unreasonable outputs in cross-year tasks, while the simulated environmental variations introduced through data augmentation provided a basis for model stability and alleviated spectral shifts caused by interannual differences. In addition, the feature selection method provided a stable and biologically meaningful structural relationship between stage temporal hyperspectral sequences and yield, thereby showing good generalization performance. By contrast, the 1D-CNN, which lacks global modeling capability, performed poorly under spectral-shift conditions. The Transformer model performed better than the 1D-CNN model in the cross-year scenario, and its global modeling capability showed a clear advantage in capturing important spectral features related to wheat yield formation. However, its performance was still noticeably inferior to that of the proposed model.

SVR and RF were more likely to learn local features tied to the training year in cross-year tasks, making it difficult for them to fully capture deeper features with stronger temporal characteristics, and thus limiting their generalization ability. Among all models, the 1D-CNN exhibited the largest fluctuations in evaluation metrics under this task, indicating that it was unable to adapt to global shifts caused by environmental variation and growth-stage changes. In contrast, the Transformer model still maintained relatively good stability in cross-year tasks, with performance close to that observed in non-cross-year experiments. Comparatively, the method proposed in this study improved model stability jointly at the data level, feature level, and task level, and achieved the best results under both cross-year settings.

To further evaluate the cross-year transferability of the proposed model, an additional independent-year validation experiment was conducted using 72 samples collected during the 2025–2026 growing season. These samples were not involved in data augmentation, feature selection, normalization-parameter estimation, model training, or hyperparameter optimization. The independent dataset contained 36 conventional-yield samples, 22 high-yield samples and 14 low-yield samples. The proposed ST-LSTM model achieved an accuracy, precision, recall, and F1-score of 0.778, 0.766, 0.814, and 0.778, respectively, on the 2025–2026 independent dataset. For the regression task, the model achieved an R² of 0.61, with RMSE and MAE values of 1122.15 kg/ha and 981.72 kg/ha, respectively. Compared with the ten-fold cross-validation results, the model performance decreased on the independent-year dataset. Nevertheless, the proposed model still retained a certain predictive ability under cross-year conditions.

## 4 Discussion

### 4.1 Mechanistic interpretation of the effects of data augmentation on yield prediction

The improvement in model performance brought by data augmentation may not be attributed solely to the increase in training sample size, but more importantly to the way in which it alters the model’s learning of informative patterns. Compared with the model trained without data augmentation (as shown in **Table 5**.), the augmented model showed a reduction in RMSE from 1101.26 kg/ha to 610.53 kg/ha and in MAE from 871.59 kg/ha to 525.21 kg/ha, while R² increased from 0.61 to 0.73. These results indicate that data augmentation substantially improved model generalization under conditions characterized by high dimensionality, limited sample size, and cross-year environmental disturbances. Yield prediction depends strongly on the model’s ability to learn robust temporal signals under complex environmental variation (Liu et al., 2026). Its contribution lies not merely in enlarging the training set, but more critically in reasonably extending the input sample distribution, thereby reducing the model’s overreliance on incidental patterns and local features contained in limited observations. As a result, the model is encouraged to focus more on the stable relationships between spectral information and yield that persist across years and environments, thus enhancing its adaptability to complex scenarios. The framework improved model reliability by capturing growth-stage-sensitive information instead of merely fitting static local patterns (Li et al., 2025).

**Table 5:** Comparison of results with different data augmentation strategies.

Model	RMSE	MAE	R²
no DA	1101.26	871.59	0.61
spectral-level	986.21	892.12	0.63
stage-level	787.55	679.72	0.69
all DA	610.53	525.21	0.73

Analysis of different augmentation strategies revealed substantial differences in their effects. spectral-level augmentation only increased R² slightly from 0.61 to 0.63, suggesting a relatively limited gain. Its primary role appears to be improving model robustness to observational noise, illumination variation, and locally abnormal bands. In essence, it mainly alleviates the model’s dependence on certain local band patterns or single-observation characteristics, thereby strengthening resistance to disturbances at the data acquisition level, but without fully exploring deeper information related to the yield formation process. The model stability is limited not only by data quantity, but also by how well the model accounts for temporal and environmental uncertainty (Ma et al., 2021). In contrast, after the introduction of stage-level augmentation, model R² increased from 0.61 to 0.69, indicating that this strategy was more critical for yield prediction. stage recombination and temporal interpolation not only increased sample size, but also expanded the distribution space of cross-stage patterns that the model could learn. To some extent, this encouraged the model to capture common features that stably reflect yield levels across varieties and environmental conditions. A similar mechanism has been suggested in knowledge-guided crop modeling studies, where sparse observations become more informative when temporal structure and agronomic consistency are incorporated into learning (Han et al., 2025). In addition, augmenting only the top and bottom 20% of yield samples helped alleviate class imbalance among high-yield, low-yield, and conventional varieties, thereby improving the stability of the decision boundary and the model’s ability to distinguish inter-sample differences.

Although the stage recombination strategy adopted in this study reduced the risk of label mismatch through class-consistency and extreme-yield constraints, the generated samples still do not represent growth trajectories that actually occurred in the field. Future work could further constrain the temporal augmentation process by incorporating climate conditions, phenological stages, leaf area index, nitrogen accumulation, or other growth-related indicators. Such improvements would make augmented samples more physiologically consistent, thereby enhancing both model performance and interpretability (Demissie et al., 2026; Cao et al., 2026).

### 4.2 Stable sensitive spectral structures revealed by repeated band selection

Based on 50 repeated scoring experiments, the analysis of frequently selected bands showed that many adjacent bands were repeatedly chosen. Although these neighboring bands may convey similar spectral meanings, their information weights may not be exactly the same across varieties with different yield levels. This phenomenon is consistent with earlier hyperspectral feature-selection studies showing that adjacent bands are often highly correlated, but still cannot be treated as completely interchangeable when the target signal is subtle and high-dimensional (Li et al., 2011). For this reason, a convolutional structure was introduced prior to band selection. The encoded features not only represent smoothed local correlations, but also serve as a re-expression of highly frequent sensitive spectral regions. Meanwhile, the convolution kernel size was fixed at 8, corresponding to the maximum width of neighboring bands identified across repeated experiments. This setting ensured that redundancy after convolution was minimized while the maximum amount of neighborhood information was preserved. Such a design is conceptually consistent with region-aware band-selection methods, which also emphasize that effective hyperspectral reduction should consider local structure rather than treating each band as an isolated variable (Wang et al., 2022).

After scoring the convolved features, some overlap among the selected high-frequency band ranges was still observed. This phenomenon may suggest that yield-sensitive spectral information is not concentrated in only a few discrete central wavelengths, but rather distributed across several continuous sensitive regions with a certain bandwidth. Similar arguments have been raised in redundancy-detection studies, where local autocorrelation and moving-window analysis showed that the informative structure of hyperspectral data often lies in contiguous spectral neighborhoods rather than in entirely independent single bands (Li J. et al., 2022). Therefore, these local continuous band ranges with finite width were retained. The repeated selection of such band ranges across random initializations further indicates that the identified features were not incidental local optima, but more likely correspond to effective spectral bands stably associated with the yield formation process (Zhang et al., 2024).

**Figure 10:**
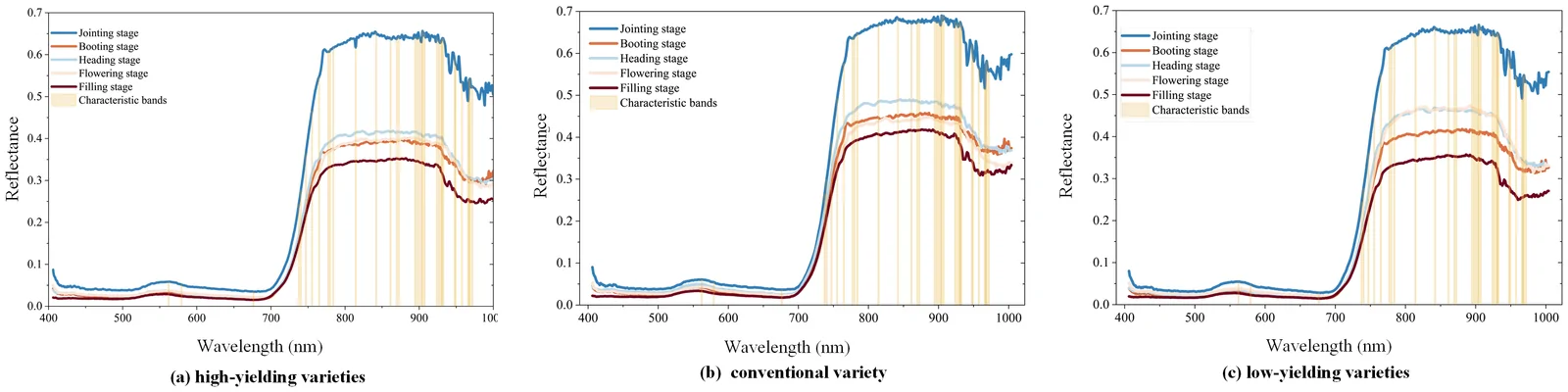
Spectral reflectance curves at critical growth stages and dimensionality-reduced feature bands for **(a)** high-yield, **(b)** conventional, and **(c)** low-yield wheat varieties.

The final set of 32 retained bands covered most of the characteristic bands identified across different varieties (**Figure 10**). This implies that yield formation is governed primarily by multiple shared physiological processes, and that the model repeatedly locks onto common features across varieties rather than completely separate sets of variety-specific bands. However, slight shifts in local peak positions indicate that different yield levels do not respond to these common spectral regions in a fully synchronized manner. This may suggest that the spectral characterization of yield formation is not centered on single isolated bands, but rather on how the response centers of high-frequency band ranges shift among varieties. In other words, the difference between high-yield and low-yield groups may not mainly manifest as a complete replacement of characteristic bands, but rather as a shift in the response focus within highly frequent spectral regions (Tan et al., 2025; Phaneendra Kumar et al., 2024; Bai et al., 2026). Overall, the feature selection framework established through 50 repeated screenings, overlap-distribution constraints, and convolution-based continuity modeling effectively shifted the objective from identifying a single optimal band combination to recognizing stable sensitive spectral structures. This interpretation is further supported by recent grouping-based convolution studies, which showed that spectral grouping can better preserve local neighborhood information than purely discrete band selection (Liu et al., 2025).

### 4.3 Joint effects of multitask learning and yield constraint on model optimization

The joint effects of multitask learning and the yield constraint not only changed the prediction results themselves, but also reshaped the optimization pathway of the model. Without such a constraint, the model can exploit a relatively large degree of freedom to fit local patterns in the training samples, some of which may be statistically meaningful but not necessarily stable or transferable. After introducing the yield constraint, the model is required not only to minimize empirical error, but also to maintain consistency between the regression output and the yield-level structure learned from the classification task. At the same time, the auxiliary supervision provided by the yield-level classification task further strengthens phenotypic information in the shared representation.

The effect of the proposed yield constraint may not be directly reflected in a substantial improvement in average accuracy metrics, but rather in the regulation of the prediction distribution. Without such a constraint, the model may produce unrealistically high or low predictions when fitting local sample differences. By contrast, the proposed constraint penalizes deviations from class-consistent yield intervals in a soft manner, so that while local spectral and temporal features are still used for regression, the overall output distribution remains more consistent with the yield-level structure of the training data. Accordingly, the main contribution of this constraint may not lie merely in reducing mean error, but in decreasing predictions that deviate markedly from realistic yield levels and in improving the stability of the output distribution across repeated experiments (He et al., 2023). Compared with a modest reduction in average error, whether the predictions remain within a practically acceptable production range is more important for variety screening.

The proposed yield constraint can also alleviate modeling bias caused by imbalanced sample distributions in the training data. In real field experiments, yield samples are usually concentrated in the middle-yield interval, whereas extremely high- or low-yield samples are relatively scarce. Under such circumstances, a single regression objective may bias the model toward densely sampled regions or cause unstable fitting due to the influence of marginal samples. After multitask learning is introduced, the yield-level classification task provides phenotypic stratification information for the shared representation, while the yield constraint further regularizes the prediction space by incorporating class-related yield priors into model optimization. Their joint effect allows model optimization to become less dominated by sample frequency and to preserve, to some extent, the relative structure among different yield levels. By suppressing unreasonable extrapolation tendencies, the constraint improves prediction balance across different yield strata. However, because the class-specific yield intervals are derived from the training-set distribution, the effectiveness of this constraint still depends on the representativeness of the training data. If the model is applied to varietal combinations or growth environments beyond those represented in the current dataset, the class-related yield priors may become less adequate for characterizing truly extreme samples. Therefore, maintaining both prediction flexibility and biological plausibility remains an important challenge for future cross-environment generalization studies.

## 5 Conclusion

The study addressed the insufficient utilization of information from different growth stages in winter wheat yield prediction, as well as the limited stability of models under complex environmental conditions. A multitask stage temporal long short-term memory network model was proposed. Data augmentation was conducted at both the spectral and temporal levels, and wheat growth trend information was incorporated into the feature selection process. In this way, the limited sample size was expanded while the growth characteristics of wheat at each growth stage were preserved. Multitask learning was combined with growth-stage temporal modeling. While characterizing the continuous variation process of winter wheat across different growth stages, the yield-level classification task was introduced to improve the model’s ability to distinguish differences among different wheat yield levels. In addition, a yield constraint was introduced as an auxiliary regularization mechanism, so that the predicted yield remained more consistent with the latent structure of yield levels, thereby further improving yield prediction performance.

Compared with models that focus only on a single task or conventional temporal relationships, the proposed model showed clear advantages in classification accuracy and prediction performance. It achieved an accuracy of 0.87 in the classification task. In the prediction task, it achieved an R² of 0.73, with RMSE and MAE values of 610.53 kg/ha and 525.21 kg/ha, respectively. Meanwhile, the model maintained good robustness in cross-year prediction. This indicates that the model is not only applicable to yield prediction under the current data conditions, but also has certain generalization potential. Overall, this study demonstrates that the stage temporal modeling strategy based on multitask learning can effectively improve the accuracy, stability, and interpretability of winter wheat yield prediction, providing new methodological support for the application of UAV hyperspectral technology in yield prediction. Future research will conduct cross-regional validation using multi-site and multi-environment datasets and explore more flexible prior-constraint mechanisms to further evaluate and improve the model’s spatial transferability, prediction accuracy, and practical applicability.

## Data Availability

The raw data supporting the conclusions of this article will be made available by the authors, without undue reservation.
